# MIND–BODY ACTIVITY SHARED MEDICAL VISIT PROGRAM FOR UNDERSERVED OLDER ADULTS WITH CHRONIC PAIN

**DOI:** 10.1093/geroni/igae098.0873

**Published:** 2024-12-31

**Authors:** Ana-Maria Vranceanu, Kate McDermott, Natalia Giraldo Santiago, Julie Brewer, Clara Vonderheide, Elizabeth Allen, Jonathan Greenberg, Christine Ritchie

**Affiliations:** Massachusetts General Hospital, Boston, Massachusetts, United States; CHOIR MGH, Boston, Massachusetts, United States; CHOIR-CASI, Boston, Massachusetts, United States; CHOIR MGH, Boston, Massachusetts, United States; CHOIR MGH, Boston, Massachusetts, United States; Massachusetts General Hospital, Boston, Massachusetts, United States; CHOIR MGH, Boston, Massachusetts, United States; Mass General Hospital and Harvard Medical School, Boston, Massachusetts, United States

## Abstract

Older adults with chronic pain in underserved community health centers have high rates of chronic pain and lack of access to evidence based behavioral interventions. With funding from an NIA/NINDS HEAL grant, we used qualitative methods, the Socioecological Model and Aaron’s implementation framework to culturally tailor an evidence based, group, mind-body activity program to meet the needs of English and Spanish speaking older adults with chronic pain and to be delivered by clinicians typically available in underserved community health centers (social workers, nurse practitioners or psychologists) and bundled within billable shared medical visits. This talk will briefly discuss our process of cultural adaptation, training of providers, and process for incorporation of the program within billable medical visits. Next, we will present results of an open pilot effectiveness trial (English and Spanish) of the tailored interventions (N=5 groups, N= 32 patients) delivered by psychologists (1 group; English), nurse practitioner (2 groups; English) and social worker (2 groups; Spanish). Feasibility markers (recruitment 86.8%; acceptability 93.9%; fidelity 95%) were excellent. We observed significant improvement in outcomes (physical function p<.001; 6-minute walk test p<.002; pain p<.001; depression p<.001) and mechanisms (pain catastrophizing p<.002; mindfulness p<.006; kinesiophobia p<.005). Qualitative exit interviews confirmed high satisfaction with the program and revealed important consideration for a future hybrid effectiveness-implementation trial.

